# Editorial: Lifestyle intervention approaches in prediabetes or diabetes

**DOI:** 10.3389/fendo.2024.1341674

**Published:** 2024-02-09

**Authors:** Othmar Moser, Susanne Kaser, Harald Sourij

**Affiliations:** ^1^ Exercise Physiology and Metabolism, Institute of Sports Science, University of Bayreuth, Bayreuth, Germany; ^2^ Division of Endocrinology and Diabetology, Department of Internal Medicine, Medical University of Graz, Graz, Austria; ^3^ Interdisciplinary Metabolic Medicine Trials Unit, Medical University of Graz, Graz, Austria; ^4^ Department of Internal Medicine I, Medical University of Innsbruck, Innsbruck, Austria

**Keywords:** exercise, diabetes, nutrition, lifestyle, physical activity

In the therapy and treatment of diabetes mellitus, physical activity, exercise and nutrition play an important role to improve disease-specific outcomes (1–3). In previous research it was shown that those with type 1 diabetes, who are more physically active face lower risk of overweight, obesity and hypertension when compared against their physically inactive counterparts (4). Furthermore, even a short-term moderate-intensity endurance training program was shown to ameliorate glycated hemoglobin (HbA_1c_) levels and functional capacity (5). At the same time, physical activity and exercise increase the risk of acute and post-exercise hypoglycemia (6) based on different pathophysiological factors (7), hindering people with type 1 diabetes to engage in regular physical activity due to the fear of glycaemic disturbances (8). In people with type 2 diabetes, physical activity and exercise where shown to improve glycaemia (9), decrease the risk of complications and comorbidities (1). Furthermore, in people with pre-diabetes, a holistic lifestyle intervention aiming to increase physical activity levels and lower the daily caloric intake, showed a 58% decreased incidence of type 2 diabetes compared with the placebo group, which was even higher than the decrease observed with metformin therapy (31% reduction) (10). In a different study, assessing the efficacy of an lifestyle intervention on improvements of short term type 2 diabetes (<3 years), it was shown that 61% of participants were able to reach disease remission, which was not observed within the standard of care control group (11).

Taking this evidence into account, there are clear recommendations from the American Diabetes Association (ADA) in regards to physical activity and exercise in people with diabetes (12): 150 min/weeks of moderate intensity exercise or 75 min/week of high-intensity exercise to improve insulin sensitivity, lung function and cardiac output and to lower the risk of cardiovascular disease; two to three strength training sessions per week to ameliorate body composition, bone mineral density, blood lipid profiles and overall cardiovascular health; flexibility/balance training (2-3 sessions per week, especially for older people with diabetes) to improve the range of motion, balance and gait and reduce the risk of falls. When focusing on nutrition therapy in people with diabetes and pre-diabetes, it is recommended in a consensus report that a variety of eating patterns are acceptable for disease management (13). In more detail, people with diabetes are recommended to emphasize non starchy vegetable, minimize added sugars and refined grains, and choose whole foods over highly processed foods to the extent possible. Additionally, specified diet interventions like fasting, low carb or low-fat diets have shown different advantages that need to be individualized based on the patients’ need (14–16).

Recapitulating, it might be misleading targeting physical activity/exercise OR nutrition therapy – the holistic approach considers both: a balanced intervention of physical activity/exercise AND nutrition therapy. This Research Topic of *Lifestyle Intervention Approaches in Prediabetes or Diabetes* covers diverse topics of lifestyle interventions for people with prediabetes and diabetes to improve therapy management and the quality of life.

Liu et al. discussed in their study “Relationship between lipid accumulation product and new-onset diabetes in the Japanese population: a retrospective cohort study” the association of obesity indicators and diabetes. The findings in this study revealed that each unit increase in lipid accumulation product was associated with a 76.8% increase in the risk of developing diabetes. Very important to note, after adding classical predictors to the model like exercise, the lipid accumulation product is superior to the “A Body Shape Index” but was not superior to other indicators in direct comparison. The conclusion drawn by Liu et al. showed that high levels of lipid accumulation product correlate closely with type 2 diabetes and are a significant risk factor for type 2 diabetes, especially in women, those with fatty liver and current smokers.

In the study by Patil et al. it was detailed that India is experiencing an epidemic of type 2 diabetes. Different factors like overnutrition and physical inactivity may contribute to these extensively increasing numbers in type 2 diabetes. Hence, the authors assessed the influence of undernutrition on the risk of type 2 diabetes among rural adolescent girls. In their study including 1,520 adolescent girls, it was shown that the prevalence of underweight was 28.8%, and stunting was seen in 30.4% of participants. Underweight and obesity using BMI were observed in 58.4% and 4.2%, respectively and the median body fat percentage was 22.5%, and excess body fat (>35%) was observed in 5.7%; furthermore, prevalence of prediabetes was as high as 39.4%. The authors concluded that diabetes screening programs are required especially for the undernourished populations.

In the study by Zhou et al., it was investigated if plant-based diets rich in flavonoids, which possess properties such as scavenging free radicals and exerting both anti-inflammatory and antioxidant effects, may reduce the risk of developing type 2 diabetes. It was found that the prevalence of type 2 diabetes was inversely associated with the intake of total flavonoids in the second quartile [Odds Ratio (OR) 0.78 95% confidence interval (CI) (0.63, 0.97), p = 0.028], in the third quartile [0.76 (0.60, 0.97), p = 0.031], and in the fourth quartile [0.80 (0.65, 0.97), p = 0.027].

In last paper of this Research Topic O’Brien et al. assessed if ADA recommendations for screening of prediabetes and diabetes (dysglycemia) within the age group 35, or younger than 35 years among adults with overweight or obesity and other risk factors, holds also true when differentiated by means of sociodemographic subgroups. In total, ADA screening criteria showed high sensitivity [95.0% (95% CI=92.7-96.6)] but low specificity [27.1% (95% CI=24.5-29.9)], which did not differ by race or ethnicity. Furthermore, sensitivity was higher among women [97.8% (95% CI=96.6-98.6)] than men [92.4% (95% CI=88.3-95.1)].

To conclude, this Research Topic showed the importance of lifestyle assessment and interventions in people prediabetes and diabetes to lower the burden on health care systems on a global level.

## Author contributions

OM: Writing – original draft, Writing – review & editing. SK: Writing – original draft, Writing – review & editing. HS: Writing – original draft, Writing – review & editing.

## References

[1] Davies MJ Aroda VR Collins BS Gabbay RA Green J Maruthur NM. Management of hyperglycemia in type 2 diabetes, 2022. A consensus report by the american diabetes association (ADA) and the European association for the study of diabetes (EASD). Diabetes Care (2022) 45:2753–86. doi: 10.2337/dci22-0034 PMC10008140 36148880

[2] Riddell MC Gallen IW Smart CE Taplin CE Adolfsson P Lumb AN. Exercise management in type 1 diabetes: a consensus statement. Lancet Diabetes Endocrinol (2017) 5:377–90. doi: 10.1016/S2213-8587(17)30014-1 28126459

[3] Moser O Riddell MC Eckstein ML Adolfsson P Rabasa-Lhoret R van den Boom L. Glucose management for exercise using continuous glucose monitoring (CGM) and intermittently scanned CGM (isCGM) systems in type 1 diabetes: position statement of the European Association for the Study of Diabetes (EASD) and of the International Society for Pediatric and Adolescent Diabetes (ISPAD) endorsed by JDRF and supported by the American Diabetes Association (ADA). Pediatr Diabetes (2020) 21:1375–93. doi: 10.1111/pedi.13105 PMC7702152 33047481

[4] Bohn B Herbst A Pfeifer M Krakow D Zimny S Kopp F. Impact of physical activity on glycemic control and prevalence of cardiovascular risk factors in adults with type 1 diabetes: A cross-sectional multicenter study of 18,028 patients. Diabetes Care (2015) 38:1536–43. doi: 10.2337/dc15-0030 26015557

[5] MüllerAMoserOSternadCAzizFUntereggerCKojzarH. Effects of 8 weeks of aerobic endurance training on functional capacity and metabolic variables in people with type 1 diabetes: A secondary outcome analysis of the ULTRAFLEXI-1 study. Diabetes Obes Metab (2023). doi: 10.1111/dom.15245 37580976

[6] Moser O Müller A Aberer F Aziz F Kojzar H Sourij C. Comparison of insulin glargine 300 U/mL and insulin degludec 100 U/mL around spontaneous exercise sessions in adults with type 1 diabetes: A randomized cross-over trial (ULTRAFLEXI-1 study). Diabetes Technol Ther (2023) 25. doi: 10.1089/dia.2022.0422 36516429

[7] Riddell MC Gal RL Bergford S Patton SR Clements MA Calhoun P. The acute effects of real-world physical activity on glycemia in adolescents with type 1 diabetes: The type 1 diabetes exercise initiative pediatric (T1DEXIP) study. Diabetes Care (2023). doi: 10.2337/dc23-1548 37922335

[8] Parent C Lespagnol E Berthoin S Tagougui S Heyman J Stuckens C. Barriers to physical activity in children and adults living with type 1 diabetes: A complex link with real-life glycemic excursions. Can J Diabetes (2023) 47:124–32. doi: 10.1016/j.jcjd.2022.10.006 36411182

[9] Schwingshackl L Missbach B Dias S König J Hoffmann G. Impact of different training modalities on glycaemic control and blood lipids in patients with type 2 diabetes: a systematic review and network meta-analysis. Diabetologia (2014) 57:1789–97. doi: 10.1007/s00125-014-3303-z 24996616

[10] Knowler WC Barrett-Connor E Fowler SE Hamman RF Lachin JM Walker EA. Reduction in the incidence of type 2 diabetes with lifestyle intervention or metformin. N Engl J Med (2002) 346:393–403. doi: 10.1056/NEJMoa012512 11832527 PMC1370926

[11] Taheri S Zaghloul H Chagoury O Elhadad S Ahmed SH El Khatib N. Effect of intensive lifestyle intervention on bodyweight and glycaemia in early type 2 diabetes (DIADEM-I): an open-label, parallel-group, randomised controlled trial. Lancet Diabetes Endocrinol (2020) 8:477–89. doi: 10.1016/S2213-8587(20)30117-0 32445735

[12] Colberg SR Sigal RJ Yardley JE Riddell MC Dunstan DW Dempsey PC. Physical activity/exercise and diabetes: A position statement of the american diabetes association. Diabetes Care (2016) 39:2065–79. doi: 10.2337/dc16-1728 PMC6908414 27926890

[13] Evert AB Dennison M Gardner CD Timothy Garvey W Karen Lau KH MacLeod J. Nutrition therapy for adults with diabetes or prediabetes: A consensus report. Diabetes Care. (2019) 42:731–54. doi: 10.2337/dci19-0014 PMC7011201 31000505

[14] Herz D Haupt S Zimmer RT Wachsmuth NB Schierbauer J Zimmermann P. Efficacy of fasting in type 1 and type 2 diabetes mellitus: A narrative review. Nutrients (2023) 15. doi: 10.3390/nu15163525 PMC10459496 37630716

[15] Moser O Eckstein ML Mueller A Tripolt NJ Yildirim H Abbas F. Impact of a single 36 hours prolonged fasting period in adults with type 1 diabetes – A cross-over controlled trial. Front Endocrinol (Lausanne) (2021) 12:656346. doi: 10.3389/fendo.2021.656346 34295305 PMC8292020

[16] Obermayer A Tripolt NJ Pferschy PN Kojzar H Aziz F Muller A. Efficacy and safety of intermittent fasting in people with insulin-treated type 2 diabetes (INTERFAST-2)-A randomized controlled trial. Diabetes Care (2023) 46:463–8. doi: 10.2337/dc22-1622 PMC9887629 36508320

